# Astaxanthin from Crustaceans and Their Byproducts: A Bioactive Metabolite Candidate for Therapeutic Application

**DOI:** 10.3390/md20030206

**Published:** 2022-03-12

**Authors:** Vida Šimat, Nikheel Bhojraj Rathod, Martina Čagalj, Imen Hamed, Ivana Generalić Mekinić

**Affiliations:** 1 University Department of Marine Studies, University of Split, R. Boškovića 37, HR-21000 Split, Croatia; martina.cagalj@unist.hr; 2 Department of Post Harvest Management of Meat, Poultry and Fish, PG Institute of Post Harvest Management (Balasaheb Sawant Konkan Krishi Vidyapeeth, Dapoli), Killa-Roha, Dist. Raigad 402 116, Maharashtra State, India; nikheelrathod310587@gmail.com; 3 Department of Biotechnology and Food Science, NTNU—Norwegian University of Science and Technology, 7491 Trondheim, Norway; imen.hamed@ntnu.no; 4 Department of Food Technology and Biotechnology, Faculty of Chemistry and Technology, University of Split, R. Boškovića 35, HR-21000 Split, Croatia; gene@ktf-split.hr

**Keywords:** astaxanthin, crustaceans, byproducts, biological activities, health benefits

## Abstract

In recent years, the food, pharma, and cosmetic industries have shown considerable interest in bioactive molecules of marine origin that show high potential for application as nutraceuticals and therapeutic agents. Astaxanthin, a lipid-soluble and orange-reddish-colored carotenoid pigment, is one of the most investigated pigments. Natural astaxanthin is mainly produced from microalgae, and it shows much stronger antioxidant properties than its synthetic counterpart. This paper aims to summarize and discuss the important aspects and recent findings associated with the possible use of crustacean byproducts as a source of astaxanthin. In the last five years of research on the crustaceans and their byproducts as a source of natural astaxanthin, there are many new findings regarding the astaxanthin content in different species and new green extraction protocols for its extraction. However, there is a lack of information on the amounts of astaxanthin currently obtained from the byproducts as well as on the cost-effectiveness of the astaxanthin production from the byproducts. Improvement in these areas would most certainly contribute to the reduction of waste and reuse in the crustacean processing industry. Successful exploitation of byproducts for recovery of this valuable compound would have both environmental and social benefits. Finally, astaxanthin’s strong biological activity and prominent health benefits have been discussed in the paper.

## 1. Introduction

The aquaculture and seafood industry productions are rising in line with the increased demand for seafood products. These sectors generate large amounts of byproducts that can be transformed into other products or used for the recovery of parts/byproducts from which high-value compounds could be extracted. The high-value compounds from the seafood industry byproducts include polyunsaturated fatty acids (PUFAs), bioactive peptides, chitin, collagen, pigments, marine enzymes, etc. The byproducts of the crustacean processing industry are considered especially underutilized. Approximately 15 million tons of crustaceans are captured (38%) and aquacultured (62%, mainly in Asian countries) around the world annually [1]. These products are considered a healthy diet choice as they are a good source of various nutrients. However, the yield in crustacean meat production is quite low, from 20 to 25%; thus, up to 80% of the raw materials are wasted [2]. This presents not only a serious ecological problem but also a financial burden for the producers. Organisms used for processing, such as prawns, shrimps, crabs, and lobsters, generate byproducts comprised of heads, exoskeletons, and appendages [3]. These byproducts are composed mainly of organic matter and contain valuable compounds, such as proteins, chitin, chitosan, lipids, carotenoids (pigments), and minerals [4]. The recovery of the valuable compounds from the crustacean byproducts could contribute to the new products/ingredient development and the sustainability of the whole sector as well as have environmental benefits. Carotenoid astaxanthin is a compound of great interest to researchers and to the food, feed, pharmaceutical, and nutraceutical industries. Due to its potent antioxidant properties declared to be stronger than vitamin E, vitamin C, and β-carotene, many health and therapeutic benefits [5,6,7,8,9,10,11] and possible application as a natural coloring in food and feed [12], astaxanthin has become a highly demanded metabolite. Besides, it is used as a feed ingredient in farming systems for obtaining desirable pigmentation of fish and seafood and improving their immunity [13]. Furthermore, its bioactivity has been associated with beneficial health effects for humans, namely its anticardiovascular, anti-inflammatory, and antiaging potentials as well as other favorable cosmetic benefits due to the improvement of skin moisture and elasticity [14]. In 2020, the global astaxanthin market size was estimated at USD 1371.24 million, and it is expected to grow [15]. The main source used for natural astaxanthin industrial production is freshwater microalgae *Haematococcus pluvialis*, and the cost of its production is estimated at 880 €·kg^−1^ [16]. The main producer is North America (67%), and the global nutraceutical industry is the biggest consumer of natural astaxanthin [17]. On the other hand, the market value of astaxanthin is determined by the purity of the product and ranges from 2200–6620 €·kg^−1^ to a high 13.240 €·kg^−1^ for astaxanthin from *H. pluvialis* [17]. Nguyen [18] compared the economic, environmental, and social impacts of astaxanthin production by chemical synthesis, fermentation, or isolation from algal material and reported huge differences among the costs of these processes: $2000, $2500, and >$7000 per kg of astaxanthin, respectively. Although the production by chemical synthesis is less expensive (about $1000 per kilo), and it is indicated as the most cost-effective way to obtain this astaxanthin [19], this process does not give a pure compound but a combination of different isoforms that have 20 times lower antioxidant capacity than their natural counterpart. Besides, to date, it has not been approved for human consumption [11,18]. The chemically synthesized astaxanthin is expected to retain its dominance on the market, especially in the feed industry; however, consumers’ focus on naturally extracted astaxanthin products is expected to rise [17]. There is limited information on the production price of natural astaxanthin from crustacean byproducts. It is dependent on the availability of the raw material, extraction methodology, and astaxanthin yield and purity [17]. At the industrial level, astaxanthin is extracted from krill and crustacean byproducts in China [19]. Su et al. [20] calculated that China alone generates 500,000 metric tons of shrimp byproducts and wastes 97 metric tons of natural astaxanthin ($650 million) every year. Evidently, the use of crustacean byproducts for carotenoid extraction has room for improvement and presents a great opportunity for the whole sector.

This paper reviews recent studies on shrimp/crab byproducts as valuable sources of natural astaxanthin (studies are from 2015 to present, found by the keywords search in the electronic databases Scopus and ScienceDirect). The objective is to provide an overview of the scientific knowledge on the chemical and biochemical properties of astaxanthin as well as an overview of its content in crustacean byproducts. To find the limitation of the successful exploitation of astaxanthin, we reviewed papers dealing with extraction protocols in search of the environmentally friendly process or green chemistry techniques for the recovery of this metabolite. The article characterizes the bioactive properties and health benefits of astaxanthin. Special attention has been devoted to data on astaxanthin sourced from crustaceans to identify potential improvements needed for its successful industrial exploitation.

## 2. The Structure and Division of the Carotenoids from Marine Origin

Carotenoids are a class of lipid-soluble pigments widely distributed in different photosynthetic organisms and some nonphotosynthetic bacteria and fungi, responsible for their red, orange, and yellow colors. These color features are the result of their chemical structure, primarily a long polyenic carbon chain while derivatization of their base structure, among other properties, results in different color hues and tones. They are primarily biosynthesized by plants, algae, yeasts, fungi, archaea, and eubacteria, but they can also be found in animals and humans, who absorb and deposit them through the diet and different metabolic reactions [21,22,23,24,25]. 

According to the latest available data, approximately 1204 naturally occurring carotenoids have been detected in 722 source organisms [26].

### 2.1. Chemical Structure and Classes

More than 95% of all known carotenoids are formed by isoprene units (C5 blocks), and based on the number of carbons in their structure, they are classified as C30 (6 isoprenoid units), C40 (8 isoprenoid units), C45 (9 isoprenoid units), and C50 (10 isoprenoid units). The class of C40 is the most abundant in nature as it is synthesized by eukaryotic organisms, bacteria, and archaea. On the other hand, carotenoids with the C30 and C50 structures are synthesized by bacteria and archaea only while those with the C45 structure are synthesized only by some bacteria. The presence of some apocarotenoids, whose normal C40 backbones have been shortened from one and/or both ends, has also been reported. Some of the well-known apocarotenoids are bixin (C25) and crocetin (C20). In some cases, vitamin A is also considered an apocarotenoid since symmetric cleavage of β-carotene gives two equivalent retinal molecules [22,27].

According to the carotenoid-structural features, they are classified into two major classes:

(a) carotenes—linear hydrocarbons that can be cyclized at the ends (e.g., β-carotene and lycopene), and
(b) xanthophylls—derivatives of carotenes with one or more oxygen-containing functional groups (oxygenated carotenoids) (e.g., zeaxanthin, lutein, capsanthin, violaxanthin, neoxanthin) [11,22,28,29].

**Figure 1:**
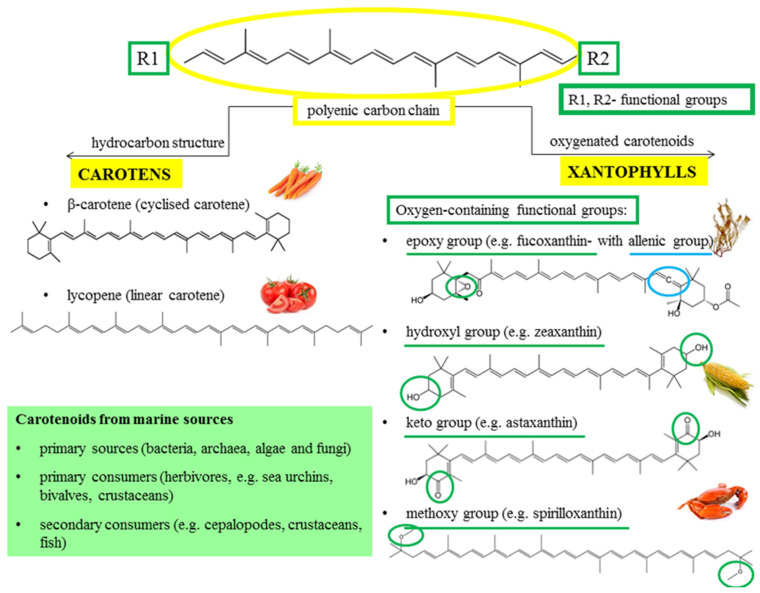
Chemical structures and sources of carotenoids.

The functional groups of xanthophylls, such as epoxy groups in neoxanthin, violaxanthin, and fucoxanthin, hydroxyl groups in zeaxanthin and lutein, the keto group in astaxanthin, and the methoxy functional group present in spirilloxanthin, are generated by the enzymatic reactions. These functional groups affect the compound solubility (higher polarity) as well as their other chemical and biological functions [22,28,30] (Figure 1).

In nature, carotenoids are found in free forms or esterified with fatty acids (compounds with hydroxyl groups, especially xanthophylls), sugars (crocetin), or proteins (carotenoproteins), which result in increasing their lipophilicity [22,24]. 

In their polyene chain, some of them contain allenic or acetylenic functional groups, like fucoxanthin and peridinin (allenic carotenoids) or crocoxanthin (acetylene carotenoid) [22]. Most of the naturally present carotenoids are in a more stable *trans* form despite the presence of multiple double bonds in their structure [22,23].

Their structural diversity is responsible for numerous biochemical and physiological functions associated with this class of compounds.

### 2.2. Carotenoids from Marine Organisms

Carotenoids are essential pigments, structural and functional components in numerous aquatic organisms (primary carotenoids), or generated after exposure to various conditions (secondary carotenoids) [31,32]. The primary sources of carotenoids are photo-autotrophic organisms; they can be found in their primary consumers (herbivores, e.g., sea urchins, bivalves, crustaceans, and fish) or in secondary consumers (e.g., cephalopods, crustaceans, fish) [30] where they are absorbed directly or modified through the metabolic reactions.

The most important carotenoids from marine organisms are astaxanthin (found in green algae, bacteria, yeast, sea snails, sea urchin, crabs, shrimps, lobsters, shellfishes, starfish, jellyfish, etc.); fucoxanthin (in seaweeds, diatoms, corals, sea urchin, starfish, etc.); β-carotene (in microalgae, seaweeds, shellfish, cyanobacteria, sea urchin, starfish, etc.); lutein (in microalgae, seaweeds, corals, shellfishes, etc.); siphonaxanthin (in green algae); mytiloxanthin (in tunicates, mussels, oysters, etc.); zeaxanthin (in microalgae, seaweeds, corals, shellfish, etc.); saproxanthin and myxol (in bacteria from the family Flavobacteriaceae); halocynthiaxanthin (in sea squirt and sea pineapple); violaxanthin, neoxanthin, and antheraxanthin (in various seaweeds); isorenieratene, renieratene, and renierapurpurin (in sponges), etc. [27,31,32,33].

## 3. Carotenoids from Crustaceans

The composition of crustacean shells varies with species and seasons; however, it is mainly composed of chitin (15–40%), protein (20–40%), calcium carbonate (20–50%), and lipids (0–14%) with a high content of omega-3 fatty acids, pigments, and other minor components [34,35,36]. These compounds have shown great bioactivity and versatility in applications. Proteins can be used as fertilizer and animal feed and calcium carbonate as a fertilizing material, filler, or white pigment [37] while chitin utilization ranges from the production of biomaterials, food, pharmaceuticals, and cosmetics to water treatments [38,39,40]. 

In crustaceans, body coloration depends on the specific pigments present in the principal layer of their exoskeleton and the subepidermal chromatophores. Among them, the carotenoids are the most significant, and the most prevalent carotenoid in commercially important crustaceans is astaxanthin [19,20]. 

### 3.1. Astaxanthin from Crustaceans

#### 3.1.1. Structure and Biochemistry

Astaxanthin (C_40_H_52_O_4_, 3,3-dihydroxy-β,β-carotene-4,4-dione), an orange-reddish-colored pigment, is widely distributed among the seafood byproducts, so it is often called marine carotenoid [41,42]. Structurally, it is a ketocarotenoid from the group of xanthophylls [7,43], with hydroxyl (OH) and keto (CO) groups at each terminal. This unique chemical structure is responsible for its characteristics and functions (Figure 1) [7,44]. Astaxanthin has two chiral carbons at the 3 and 3′ positions, which allow the formation of various isomers and stereoisomers [20,45,46]. The three main isomers of astaxanthin are two enantiomers (3S, 3′S), (3R, 3′R) and one mesomer (3R, 3′S), depending on the spatial orientation of hydroxyl groups in the chiral carbons [47]. The stereoisomers (3S, 3′S) and (3R, 3′R) are the most abundant in nature [48]. Astaxanthin also occurs in two geometrical isomers, *trans*- and *cis*- (E and Z), depending on the configuration of the double bonds in the polyene chain [49]. 

Astaxanthin is derived from the β-carotene or zeaxanthin by β-carotene hydroxylase and β-carotene ketolase, respectively [50]. In crustaceans, it can be found unesterified (free) or esterified (mono- and diesterified) with various fatty acids [51]. Its structure has a significant impact on its bioavailability, as suggested by Yang et al. [46], who investigated the stability and bioavailability of different astaxanthin derivates (free, mono-, and diesters) by in vitro and in vivo digestion models and concluded that esters with long-chain and saturated fatty acids are the most stable and that esters containing short-chain fatty acids (esters with high unsaturation of fatty acids and monomers in comparison to the dimers) have higher bioavailability. Several studies have also reported their interactions forming lipoproteins and carotenoproteins [41,52]. Besides, a recent study by Yang et al. [43] suggested that encapsulation of astaxanthin (in the starch-based emulsion) imparted its stability and increased accessibility. The bioaccessibility of astaxanthin was found to increase from 50 to 100% by encapsulation in gelatin gel [53], imparting its stability for inclusion in food supplements or matrices. After being ingested, astaxanthin is diffused through the intestine and forms micelles, which are partially absorbed and further stored in the liver. From there, astaxanthin is transported to the tissues via a circulatory mechanism [10].

#### 3.1.2. Sources

**Figure 2:**
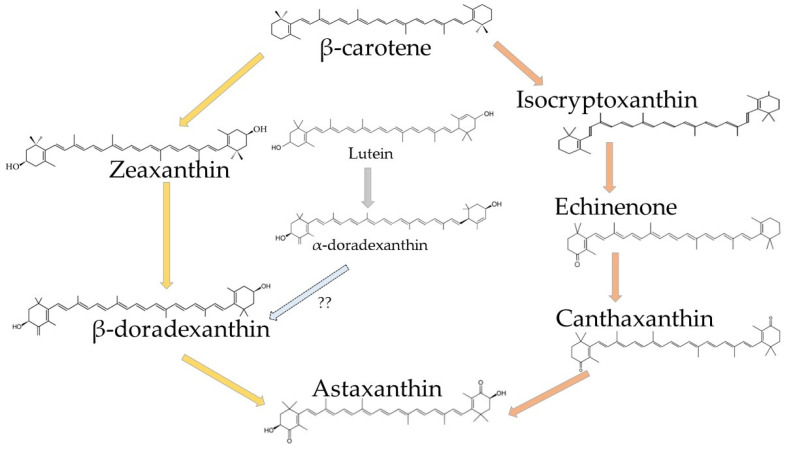
The pathways for β-carotene conversion to astaxanthin as proposed by Rhodes [54]. Orange arrows indicate the pathway proposed for most crustaceans. A light blue arrow with a dotted line is a hypothesized pathway for lutein conversion to astaxanthin; yellow arrows indicate an alternative conversion pathway proposed for crustaceans that may not rely on echinenone and canthaxanthin as intermediates.

**Figure 3:**
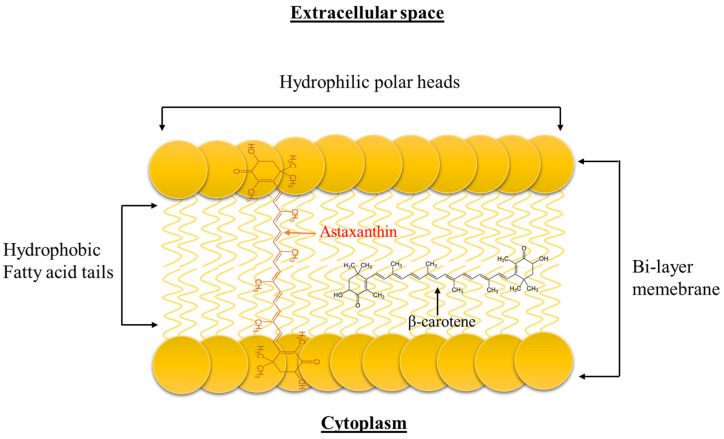
Orientation of astaxanthin in the cell membrane (adapted from Kishimoto et al. [58]).

Crustaceans can synthesize astaxanthin through the metabolic reactions from other carotenoids, such as β-carotene, lutein, and zeaxanthin, that are ingested (Figure 2), or they can just store it directly by consuming other animals that already performed the bioconversion [31]. Crustaceans accumulate astaxanthin in their tissues, cuticles, hemolymphs, and eggs, where it exists in both forms, free and/or esterified as mono- or diesters with palmitic, oleic, stearic, or linoleic acid. The free astaxanthin circulates in the hemolymph and may be incorporated in the cell membranes where it prevents lipid peroxidation and participates in maintaining membrane structure (Figure 3). On the other hand, esters are mainly stored in the tissue. The structure of astaxanthin can fit the hydrophobic polyene carbon chain inside the bilayer cell membrane while its polar terminal rings are located near its surface [19].

Amongst crustaceans, astaxanthin is widely distributed in shrimps, crawfish, crabs, lobsters, and Antarctic krill byproducts [55,56] (Table 1). In the exoskeleton of crustaceans, astaxanthin exists as carotenoproteins, such as crustacyanin, providing various colors from red, purple, blue/blue-black, to yellow [30,57]. In the complex form (i.e., associated with proteins and lipids e.g., carotenoprotein or carotenolipoprotein), it is blue to green, which, upon denaturation/separation/cleavage and the release of astaxanthin from the complex, imparts a reddish-orange color [56].

Although most of the commercialized astaxanthin originates from the green microalgae *H. pluvialis* [59], crustaceans are considered its good source, especially if it is recovered from the seafood processing industry byproducts [41,60,61,62]. These byproducts, such as the head, shell, and tail of shrimps, are rich in astaxanthin and are among the most explored sources of natural astaxanthin [4]. Besides, wastewater from shrimp cooking/processing should also be considered as its possible source [56]. A recent literature overview (data reported in the period from 2016 to 2021) of astaxanthin content from different crustaceans and crustacean byproducts is given in Table 1.

The accumulation of astaxanthin in crustaceans has been suggested to play an important role as an immune-stimulating and antioxidant agent. The crustaceans possess endogenous enzymes and nonenzymatic, exogenous compounds that can scavenge free radicals. The generation of reactive oxygen species (ROS) could be induced by high irradiation (mainly ultraviolet), oxygenic photoautotrophy, and the presence of xenobiotics. The major antioxidant enzymes able to detoxify ROS are superoxide dismutase, catalase, and peroxidase. Furthermore, crustaceans possess peroxinectins, proteins involved in the immune defenses, including peroxidase activity. Nonenzymatic, exogenous compounds with antioxidant properties are obtained through dietary intake. Nonenzymatic antioxidants can be divided into hydrophilic compounds, present in aqueous cellular compartments such as vitamin C, glutathione, and lipophilic compounds, such as carotenoids, which can be included in cell membranes and associated with lipoproteins. Hence, carotenoids can regulate immunopathology and play an important role in the modulation and the evolution of immune defenses [57,79].

#### 3.1.3. Extraction

Traditionally, astaxanthin has been extracted from crustacean byproducts by solvent extraction, oil extraction, or microbial fermentation. Due to the harsh processing conditions that characterize these conventional extraction techniques, the yield, quality, and stability of astaxanthin are meager. Considering these limitations, novel, nonthermal techniques, such as supercritical fluid extraction, high-pressure extraction, ultrasonication, and pulsed electric field, have been evaluated (Table 1) [65,77,80,81]. 

The high-pressure extraction of astaxanthin from shrimp byproducts was reported by Li et al. [74]. Solvation properties of used solvents (ethanol, acetone, dichloromethane) and pressure application (0 to 600 MPa) exhibited a high impact on astaxanthin extraction from shrimp processing byproducts. The application of high-pressure damages cellular membranes and disorders the fiber structure, leading to the higher diffusion of solvent and enhanced astaxanthin extraction. However, pressure increases over 300 MPa showed a negative impact on astaxanthin recovery.

Ultrasound application (23.6% amplitude, 26.3 °C for 13.9 min) improved the extraction of astaxanthin from shrimp shells [80]. Ultrasound-induced cavitation caused fragmentation of the shell matrix, increasing the solubilization of bioactive compounds and their extraction by solvents. Solvent polarity and extraction time had a significant impact on the extracted yield of astaxanthin. 

Supercritical fluid extraction employing different solvents is found to be an effective technique for astaxanthin extraction from crustacean byproducts. Optimized conditions (56.88 °C, 215.68 bar, and flow rate of 1.89 mL/min) yielded both free (12.20 µg/g) and conjugated (58.50 µg/g) astaxanthin [77]. This technique is dependent upon the solubility of solute, which is influenced by the temperature and pressure. Besides, solvent selection has a key role in astaxanthin extraction [82,83]. Higher concentrations of ethanol (5, 10, and 15%) resulted in a significant increase of astaxanthin yield (from 26.0 to 34.8 µg/g) [84]. Besides, the application of high pressures (>400 bar) in supercritical fluid extraction hampers its extraction. 

Microbial fermentation followed by supercritical extraction from shrimp waste liquid fraction was recently optimized [65]. Results suggested that fermentation of raw material by lactic acid bacteria improved extraction of astaxanthin in comparison to the common supercritical extraction. The fermentation increased the extraction of lipophilic compounds in the liquor and enzymolysis of shrimp shells while neutral proteases resulted in a 3.7-fold higher astaxanthin concentration (134.20 µg/g) [85]. 

The combined effects of ultrasound- and pulsed electric field-assisted treatment on the extraction of astaxanthin from shrimp byproducts were reported by Gulzar and Benjakul [52]. Disintegration by damaging the cephalothorax was found to increase with an elevation of the electric field strength. Furthermore, ultrasound caused electroporation, improving the mass transfer; thus, the recovery of astaxanthin was recorded as well. The pulsed electric field was found to be a more suitable technique than ultrasonic extraction, in terms of damage caused to the cells.

### 3.2. Biological Activity of Astaxanthin

Highly reactive oxidative molecules, like free radicals or ROS, through the reactions with various cell-regulating molecules, cause damages that induce different disorders in the human body. These reactions can be effectively inhibited by the application of endogenous and exogenous antioxidants. Among carotenoids, astaxanthin has been recognized as one of the most effective antioxidants due to its unique chemical structure and both lipophilic and hydrophilic properties [7,10,48]. The proposed mechanisms of astaxanthin antioxidant action are electron donation, bonding with free radicals to form less active forms and nonreactive products, transportation of radicals along its own carbon chain outside the cell, inhibition of ROS formation, and metal chelation by two adjacent oxygen atoms on the cyclohexene ring. All of these antioxidant activities have been reported by numerous in vitro and in vivo studies [6,8,23,45,51,86,87,88,89].

In the intense search for novel antibacterial agents that would contribute to the global health issue of bacterial antibiotic resistance that is affecting treatments of the various infections, astaxanthin has been shown as a promising candidate. Generally, the nature and mode of action of antimicrobials is unclear and/or partially explained, but it is known that one of the common antimicrobial mechanisms is based on the ability of antibacterial agents to generate diverse forms of ROS while interacting with cell targets [90,91]. The generated ROS species (e.g., peroxide radical or superoxide anion) are not as reactive as those generated through the Fenton reaction (e.g., hydroxide radical) showing the ability to cause cellular dysfunction and/or cell death [92]. This concept of antibacterial activity through ROS generation has been implicated for carotenoids, among others, and another possible antimicrobial action of astaxanthin is the inhibition of biofilm formation (antibiofilm activity), both in bacteria and fungi [93]. The antimicrobial activity of astaxanthin bacteria has been also widely reported [61,92,93,94,95,96,97,98]. Furthermore, beneficial pharmacological effects of astaxanthin, such as antioxidant, antimicrobial, anticancer, anti-inflammatory, neurodegenerative, gastrointestinal, cardiovascular, antidiabetic, ocular, and skin-protective effects, have been widely reported [6,48]. Generally, scientists are reporting that astaxanthin’s physical and chemical interactions with cell components and/or cell membranes are key features for its high effectiveness in these disorders [48,92], and some results of the novel studies (from 2015 to present) regarding these activities are reported in Table 2.

### 3.3. Health Benefits, Therapeutic Application, and Safety of Astaxanthin Application in Humans

Considering the health benefits imparted by astaxanthin due to its biological activity as discussed earlier, it is widely distributed among the food web [56]. The wide arrays of bioactivities possessed by astaxanthin from shrimp byproducts have been found to exhibit health benefits both in vitro and in vivo [126,127]. Astaxanthin reduced the release of interleukins, cycloxygenase-2 and nitric oxide generation, and DNA damage induced by radioactivity as well as improved inflammatory-related pathways [110,128,129,130,131,132,133,134]. It has been found to impart a healthy state by reducing oxidative stress, neutralizing singlet oxygen, scavenging free radicals, inhibiting lipid peroxidation, and improving immunity and muscle health [5,7,89,135,136,137]. Several studies have also indicated the ability of astaxanthin from shrimp to reduce obesity, hypertension, hyperlipidemia, and heart-related ailments [138,139,140,141]. These properties have been attributed to its anti-inflammatory properties and reduction of oxidative stress in glucose and lipid metabolism [58,142,143]. The management of blood pressure by astaxanthin was explained by its ability to reduce oxidative stress and vasoconstriction [7,141,144,145,146]. Astaxanthin from natural sources increased endurance, improved resistance to stress, and prevented inflammations and ulcers [145,147].

Astaxanthin gained popularity due to its numerous health benefits and has been proven safe for oral administration [148,149]. Considering its low bioavailability/absorption rate in its free form, it is administered in the ester- or nanoform [100,145,150]. Further, the dosage related to meal timing (before and after meal) and the form (monoester or diester) affects its concentration and availability [151,152]. Astaxanthin imparts protection against a cytokine storm controlling adversaries and pathogenesis caused by COVID-19 [153]. The ability of astaxanthin to enhance immunity by increasing the production of immunoglobulin A responsible for antibody production has also been reported [154]. In addition, an improvement in human skin health measured by skin elasticity (gross, net, and biological) due to supplementation of dietary astaxanthin with collagen hydrolysate was observed after 12 weeks of application [155].

Cardiovascular disorders are responsible for a large number of deaths worldwide [5,156], and since they are often connected with oxidative stress and ROS, astaxanthin can be used in their prevention due to its strong antioxidative activity [126,157,158,159]. Furthermore, the oral administration of astaxanthin reduced cholesterol in mice with immediate spread across the body [160]. It has also been found that astaxanthin reduces stroke incidence and myocardial infarct size [5]. Lowered/delayed oxidation of low-density lipoprotein, reduced oxidative stress, lipid metabolism, anti-inflammatory properties, and improved blood rheology are evidence of its beneficial properties against cardiovascular diseases [10,126,159,161,162,163,164,165,166]. Astaxanthin administered orally at 50 mg/kg body weight improved kidney function in mouse models [167]. Recently, neuroprotection has gained immense importance due to the increase in neurodegenerative diseases. The incidence is linked with high stress causing damage, misfolding, and aggregation leading to the death of neurons [168]. A recent review by Fakhri et al. [169] reported the neuroprotective effects imparted by astaxanthin. The neuroprotective effects of astaxanthin are related to its ability of multitarget treatment and its capability to influence signaling pathways (PI3K/Akt pathways), neuroinflammation, lipid peroxidation, and microcirculation; inhibit amyloid-β peptide, aggregation, and mitochondrial functions; and decrease cell death helpful for treating Parkinson’s and Alzheimer’s diseases as well as neuropathic pain and suppression [169,170,171,172]. The improved cognitive ability of elderly patients evaluated by improved reaction time and low rate of error in the memory-based game was detected for patients that were taking astaxanthin supplements [173]. Furthermore, improved motor skills were also observed after 12 weeks [174].

Astaxanthin from natural sources has been suggested as safe for application with no adverse (mutagenic, carcinogenic, biochemical, and hematological) effects for humans [7,142,147,158]. However, there is a lack of literature on marine astaxanthin therapeutic applications and safety concerns since it is known that supplementation of different amounts and forms (natural and synthetic) exhibits different levels of bioactivities [159,175,176,177]. Considering the safety issues, the allowed levels of astaxanthin in food supplements were 8 mg/day, and acceptable daily intake for adults ranged from 0.034 to 0.2 mg astaxanthin/kg body weight [178]. On the contrary, the highest dosage evaluated (i.e., 700–1000 mg/kg body weight) reported subchronic toxicity in rats [176,179,180]. Furthermore, the application of astaxanthin in the astaxanthin-dimethyldisuccinate form at 100 mg astaxanthin/kg completed diet (138 mg astaxanthin/kg) was recommended for effective coloring in fish and crustaceans without dermal or ocular risk. Considering this, the maximum exposure of the consumer would be 25 mg astaxanthin/kg and 4 mg astaxanthin/kg [181].

There are different applications of natural and synthetic astaxanthin. The main limitation in the industrial application of natural astaxanthin is its price; however, the nutraceutical and pharmaceutic industries use more expensive forms of astaxanthin due to the increase concerns of the consumers for their health and environment [182]. Besides a high price, limited sources of natural astaxanthin stand in the way of widening its application. So far, astaxanthin has been applied as a coloring agent in aquaculture feed [13], food industry, and various cosmetics [19]. It has been widely used as a food supplement and functional ingredient but also as an enhancing agent for food quality and nutritive value [183]. Despite the widely documented, positive effects of astaxanthin, its use in practice is still very limited.

## 4. Conclusions 

In recent years, studies showed a strong nutraceutical and therapeutic potential of natural astaxanthin. Its powerful antioxidant and other bioactive properties (anti-inflammatory, cytotoxic, antiproliferative, and anticancer activity) as well as favorable safety profile make astaxanthin a promising compound with the ability to prevent or even treat different health conditions. Among them, there is reported evidence of astaxanthin’s ability to delay cardiovascular diseases, adverse neuroprotective effects, and harmful inflammations in the organism while improving lipid and glucose metabolism. Further, astaxanthin has benefits in animal health and aquaculture production. The source of natural astaxanthin has been limited to microalgae (fungi and krill in smaller proportions). Many studies evidence the availability/yield of the astaxanthin from different parts of crustacean byproducts. The main challenge is the unknown cost-effectiveness of the production process as well as the lack of information on the amounts currently obtained from byproducts. There is also a lack of comprehensive studies on the economic aspects of astaxanthin production from crustacean byproduct at the industrial level. Besides, for the successful scale-up of the extraction methodologies and exploitation at the industrial level, there are additional challenges that need to be addressed. Information on the availability of the raw material from processing plants is needed as well as the seasonal effect on astaxanthin content. In addition, it is necessary to distinguish an environmentally friendly process or green extraction method among the promising techniques, such as microwave-assisted, supercritical extraction using CO_2_ and co-solvents or enzymatic extraction, and optimize/standardize it for testing in the industrial environment. During the extraction processes, the quality of astaxanthin extract should be considered as well as the feasibility of the process. Besides, additional work is needed to determine the structural and biological activity-related mechanisms of natural astaxanthin. Further, adequate doses and encapsulation of crustacean byproduct astaxanthin for supplement and food applications should be established considering its bioavailability as well as research to explain the biological mechanisms associated with its therapeutic potential. 

## Figures and Tables

**Table 1:** Crustaceans and their byproducts as a source of astaxanthin (review of studies from 2015–2022).

Species	Byproduct	Extraction Procedure	Astaxanthin Content	Salient Finding	Reference
Shrimps and prawns (*Litopenaeus vannamei (L.v), Macrobrachium rosenbergii (M.r), Penaeus monodon (P.m), Fenneropenaeus chinensis (F.c),* and *Penaeus japonicus (P.j)*)	Head, shell, and tail	Solvent extraction using dichloromethane: methanol (1:3, *v*/*v*)	19.2 µg/g (*L.v.*), 15.7 µg/g (*M.r.*), 2.9 µg/g (*P.m.*), 7.1 µg/g (*F.c.*), and 5.8 µg/g (*P.j.*)	The byproduct yield was 44.06–62.53%.	[63]
Brown crab *(Cancer pagurus)*	Crab shells	Microwave (MW) pretreatment in ethanol (at 140 °C and 300 W, during 90 s) followed by supercritical fluid extraction (SFE) (500 bar, 40 °C, and 13 wt % ethanol content, 30 min)	1023 µg/g dry extract	In comparison to conventional extraction, the SFE conditions after MW pretreatment gave the best results.	[60]
Shrimp (*Parapenaeus longirostris*)	Exoskeleton, including cephalothorax and abdominal parts	Extraction using fish oil (CVO) and different fatty acid ethyl esters (TFA) and by SFE (350 bar, 40 °C, 30 min of static extraction followed by dynamic extraction with a CO_2_ flow 2.5 L/min for 2 h)	CVO: 149.1 ± 0.8 µg/g TFA: 160.1 ± 8.9 µg/g	The highest astaxanthin yields were obtained for wet byproducts, extracted with ethyl esters fatty acids at a 2.0 ratio.	[64]
Shrimp (*Litopenaeus vannamei*)	Fermented shrimp exoskeleton	SFE (300 bar, 60 °C, and 6 mL/min)	12.62%, 0.52 µg/g	Extracts showed antioxidant activity in vitro.	[65]
Tiger prawn (*Penaeus monodon*) and mud crab (*Scylla serrata*)	Discards	Autolysis at 55 °C for 20 min on a hot plate with continuous stirring	35.76 ± 6.74 μg/g	The highest astaxanthin amount was found when the 60:20 shrimp:crab ratio was used.	[66]
Blue crab (*Portunus segnis*)	Shells	Conventional extraction, enzymatic extraction, Soxhlet, maceration	5045 μg/g extract	The highest amount of total carotenoid content was found for combined enzyme-assisted extraction and maceration in hexane/isopropanol (50/50; *v*/*v*).	[42]
Atlantic shrimp (*Pandalus borealis*)	Shells	UAE solvent extraction by acetone, hexane/isopropanol 3:2 (*v*/*v*), and methanol for 5 min at 25 °C	270.04, 284.48, and 57.34 mg/g	Hexane/isopropanol extraction resulted in the highest amount of extracted astaxanthin.	[67]
Shrimp (species not determined)	Shells	Degradation by *Aeromonas hydrophila*	2.14 ± 0.13 μg/ml	The optimized culture media for higher astaxanthin recovery is characterized by the following conditions: pH 7.0, monosodium glutamate 3% (*w*/*v*), glucose (1% *w*/*v*) and 30 °C.	[68]
Brown crab (*Cancer pagurus*)	Residues	Supercritical fluid extraction (500 bar, 40 °C, 30 min, 50 g/min)	5.18 µg/g	Optimized conditions yielded a 1.5-fold higher content of astaxanthin.	[69]
Pink shrimp (*Farfantepenaeus subtilis*)	Shrimp waste paste	Extraction using palm olein (90 mL/2.5 g) at 50, 60, and 70 °C	26.38 µg/g (50 °C), 28.62 µg/g (60 °C), and 29.18 µg/g (70 °C)	Extraction at 70 °C yielded 50.42% astaxanthin.	[70]
Shrimp (*Litopenaeus vannamei*)	Shells	Shrimp shells, dried under vacuum (40 °C and 175 MPa), were extracted by ethanol	28.9 µg/g	The obtained isolate exhibited high antioxidative activity, no toxic effect up to 160 µg/mL on human fibroblast cells, and anti-tyrosinase (12.2 µg/mL) properties.	[71]
Shrimps (*Parapenaeopsis sculptili*, *Metapenaeus lysianassa*, *Macrobrachium rosenbergii*, *Metapenaeopsis hardwickii*, *Penaeus merguiensis*, and *Penaeus monodon)*	Carapace	Extraction using acetone and methanol (7:3 *v*/*v*) and high-pressure processing (HPP) (210 MPa, 10 min)	46.95 µg/mL (conventional) 68.26 µg/mL (HPP)	HPP improved astaxanthin extraction by around 45%. *P. monodon* yielded the highest astaxanthin with a shorter extraction time.	[72]
Shrimp (*Procambarus clarkia*)	Shells	Extracted using ethanol (1:7) for 20 min at 50 °C using ultrasound (40 kHz) and dried under a vacuum	43.7 µg/g	Extraction using optimized conditions increased purity by 250 times, exhibiting great application abilities.	[62]
Shrimp (species not determined)	Fresh head, cooked head, fresh shell and cooked shell	Extraction by cooking at 90 °C for 15 min	3.64 mg/g (fresh head), 2.38 mg/g (cooked head), 14.65 mg/g (fresh shell), 11.76 mg/g (cooked shell)	Fresh shells contained the highest amount of astaxanthin, and cooking slightly impacted its content.	[73]
Shrimp (*Penaeus vannamei* Boone)	Shells	HPE using acetone, dichloromethane, and ethanol	Range from 42.3–72.9 μg/g depending on applied pressure and time	HPE resulted in higher extraction yield with improved antioxidant activity.	[74]
Shrimp (*Litopenaeus vannamei*)	Cephalothorax, cuticles, pleopods, and tails	Lipid extraction for 30 min with ethyl acetate (10 g/50 mL)	7 ± 1 mg/g	Valorization of shrimp byproducts by the production of an extract rich in bioactive compounds, such as astaxanthin, PUFAs, and α-tocopherol.	[75]
Blue crab (*Callinectes sapidus*)	Crab byproducts	Enzymatic hydrolysis with alcalase and bromelain	Range from 12.0–97.7 μg/g residue	Production of chitin and astaxanthin-enriched extract using enzymatic hydrolysis.	[76]
Tiger shrimp (*Penaeus monodon*)	Shrimp waste	Supercritical fluid extraction using carbon dioxide with 15% (*v*/*v*) ethanol	58.50 ± 2.62 µg/g astaxanthin and 12.20 ± 4.16 µg/g free astaxanthin	Use of modeling to determine the best extraction conditions, which were 215.68 bar, 56.88 °C, and 1.89 mL/min for 120 min.	[77]
Red (*Aristaeomorpha foliacea*) and pink shrimp (*Parapenaeus longirostris*)	Muscle and cephalothorax	Solvent extraction using Bligh and Dyer method	For *A. foliacea* of total carotenoids: 34.73 ± 0.87 (muscle) and 37.55 ± 0.64 (cephalothorax) % (*w*/*w*). For *P. longirostris* of total carotenoids: 34.32 ± 0.58 (muscle), 49.08 ± 0.82 (cephalothorax) % (*w*/*w*).	Analysis showed higher content of PUFAs (mainly omega-3) and high concentrations of carotenoids (astaxanthin followed by lutein).	[78]

**Table 2:** Biological activity of astaxanthin (review of studies from 2015–2022).

Activity	Form of Astaxanthin and Its Action	Reference
Antioxidant	Better activity of isolated astaxanthin from crabs in comparison to the standard compound investigated by scavenging activity against hydrogen peroxide and 2,2-diphenyl-1-picryl hydrazyl (DPPH) radicals, as reducing power and metal-ion-chelating ability.	[94]
	In vivo antioxidant efficiency on the alcohol-induced oxidative damage in mice of the water-dispersible, astaxanthin-rich nanopowder.	[99]
	Improved antioxidant properties of astaxanthin biopolymer nanoparticles in comparison to the free compound tested by in vitro scavenging activity against 2,2′-azino-bis(3-ethylbenzothiazoline-6-sulfonic acid) (ABTS).	[100]
	Higher antioxidant activity of microencapsulated astaxanthin from *Phaffia rhodozyma.*	[101]
	Applied supercritical emulsions extraction technology resulted in encapsulated astaxanthin in ethyl cellulose with good antioxidant activity.	[102]
	Effectiveness of astaxanthin in form of nanohydrogels in the neutralization of ROS in vitro.	[103]
Antimicrobial	The extent of ROS involvement in antibacterial activity against *S. aureus*, *B. cereus*, *P. aeroginosa,* and *E. coli*	[92]
	High activity of astaxanthin isolate from crabs against *E. coli* detected using the agar diffusion method.	[94]
	Confirmed antagonism of the astaxanthin methanolic isolate from *Sphingomonas faeni* against common food-borne pathogens.	[96]
	Good antimicrobial activity of astaxanthin from crustacean shell byproducts against *Escherichia coli*, *Bacillus*, *Staphylococcus*, and *Pseudomonas.*	[61]
	Good antimicrobial activity of astaxanthin from *Penaeus monodon* against four bacteria (*E. coli, E. aerogenes, S. aureus,* and *B. subtilis*), especially for extracts obtained by high-pressure processing.	[97]
	Effectiveness of astaxanthin from *H. pluvialis* against *E. coli, Salmonella typhi, Vibrio cholera,* and *S. aureus*).	[98]
	Astaxanthin in bioactive polymers showed significant reduction of bacterial growth and biofilm formation, especially against MRSA.	[104]
	The good activity of astaxanthin-alpha tocopherol nanoemulsions through the disruption of the integrity of the bacterial cell membrane detected by MIC, MBC, and disk diffusion methods.	[105]
	Astaxanthin from Asian tiger shrimp shell showed good activity in killing and growth inhibition of *E. coli*, *Pseudomonas aeroginosa*, *Salmonella typhi*, *S. aureus*, and *Streptococcus mutans* bacteria.	[106]
Anti-inflammatory	Effective in various diseases (diabetes mellitus, Alzheimer’s and Parkinson’s diseases, neuropathic pain, kidney-related diseases, hepatitis, dry eye disease, atopic dermatitis, and inflammatory bowel disease)	[107]
	The activity is demonstrated by recording suppression of proinflammatory cytokines and inflammatory mediator production in rats with monosodium urate crystal-induced arthritis.	[108]
	Astaxanthin alleviated the status of epilepticus-induced hippocampal injury in rats and improved cognitive dysfunction.	[108]
	The anti-inflammatory effect of orally administered astaxanthin was confirmed in mice with ovalbumin-induced asthma.	[109]
Cytotoxic, antiproliferative, and anticancer activity	Astaxanthin-alpha tocopherol nanoemulsions showed cytotoxicity as a measure of cell viability of four cell lines (CT26, HeLa, Panc1, and T24) and showed a significant decrease in viability after 1 and 2 days of exposure.	[105]
	Dose-dependent toxicity and antiproliferative effect of gold nanoparticles synthesized using astaxanthin against human breast cancer cells (MDA-MB-231).	[110]
	Microencapsulated astaxanthin showed inhibition of lipid peroxidation and significant cytostatic activity on adipose-derived stem cells.	[111]
	Oral treatment of astaxanthin nanoemulsion demonstrated a chemotherapy effect in mice with lung metastatic melanoma by triggering apoptosis.	[112]
	Astaxanthin administered intragastrically in mice with PC-3 xenograft prostate tumor significantly inhibited its growth.	[113]
	Astaxanthin suppressed the occurrence of *N*-nitrosomethylbenzylamine-induced esophageal cancer in rats through antioxidant and anti-inflammation capacity increase.	[114]
	Significant inhibition of the development of liver cell adenoma and hepatocellular carcinoma in diethylnitrosamine-treated mice by ameliorating serum adiponectin level and improving oxidative stress.	[115]
	Effect on subchronic testis injury induced by SnS_2_ nanoflowers in mice; treatment attenuates testicular ultrastructure alterations and histopathological injury and alleviated testicular inflammation, oxidative stress, apoptosis, and necroptosis.	[116]
	Astaxanthin-alpha tocopherol nanoemulsions showed wound healing potential through scratch assay on HeLa, CT26, and T24 cells.	[105]
Hepatoprotective	Astaxanthin-rich nanopowder prepared by nanoencapsulation and freeze-drying showed in vivo antioxidant effect on the alcohol-induced oxidative damage in mice, making the hepatic injury less severe.	[99]
	Astaxanthin-loaded liposomes provided therapeutic and reparative effects on mice with alcoholic liver fibrosis.	[117]
	Astaxanthin encapsulated within liposomes caused a reduction of lipopolysaccharide-induced acute hepatotoxicity in rats.	[118]
	Astaxanthin pretreatment reduces the effect of acetaminophen-induced liver injury in mice by reduction of ROS generation, inhibition of oxidative stress, and reduction of apoptosis	[119]
	Protection from pancreatic damage and reduces oxidative stress in rats with acute pancreatitis.	[120]
Antidiabetic	Significant decrease of total cholesterol and blood glucose levels and increase of high-density lipoprotein cholesterol levels in rats.	[121]
	Oral administration of astaxanthin reduced lung damage in rat pups with bronchopulmonary dysplasia (induced by hyperoxia and lipopolysaccharide).	[122]
Eye health	Protective effect against dry eye disease in vitro on human corneal epithelial cells cultures and in vivo in mice.	[123]
Skin health	Protective effects on age-related skin deterioration and environmentally induced damage.	[124]
	Liposomal astaxanthin showed antidermatotic effects in mice with phthalic anhydride-induced atopic dermatitis.	[125]

## Data Availability

Not applicable.
